# Supplementary material for: Timbral cues underlie instrument-specific absolute pitch in expert oboists
Source: PLoS One. 2024 Oct 3;19(10):e0306974. doi: 10.1371/journal.pone.0306974 (PMC11449301; doi:10.1371/journal.pone.0306974)
Supplement: S1 Table — (DOCX) [file pone.0306974.s001.docx]

**SUPPORTING INFORMATION**

**“Timbral cues underlie instrument-specific absolute pitch in expert oboists”**

Please cite as: Hansen, N. C. & Reymore, L. (2024). Timbral cues underlie instrument-specific absolute pitch in expert oboists. *PLOS ONE*.

**S1 Table.** Participant-level descriptive statistics for Experiment 1.

|  |  |  | **Mean accuracy (SD)** | | | **Mean absolute semitone error (SD)** | | |
| --- | --- | --- | --- | --- | --- | --- | --- | --- |
| **ID** | **ISAP** | **Sub-category** | ***Oboe*** | ***Piano*** | ***Oboe>Piano*** | ***Oboe*** | ***Piano*** | ***Oboe<Piano*** |
| 1 | 0 | Quasi both | 0.9375 | 0.8125 | 0.1250 | 0.4375 | 0.1250 | -0.3125 |
| 2 | 0 | Quasi ob | 0.3125 | 0.1563 | 0.1563 | 0.7500 | 1.6250 | 0.8750 |
| 3 | 1 | Quasi both | 0.6563 | 0.2188 | 0.4375 | 0.2188 | 1.1250 | 0.9063 |
| 4 | 1 | Quasi ob | 0.4688 | 0.0938 | 0.3750 | 0.4688 | 0.5938 | 0.1250 |
| 5 | 1 | Quasi both | 0.9688 | 0.6563 | 0.3125 | 0.4063 | 0.0938 | -0.3125 |
| 6 | 0 | Global AP | 0.9688 | 0.9688 | 0.0000 | 0.0625 | 0.3125 | 0.2500 |
| 7 | 0 | Quasi both | 0.7813 | 0.5938 | 0.1875 | 0.2813 | 1.0313 | 0.7500 |
| 8 | 0 | Quasi both | 0.8125 | 0.6563 | 0.1563 | 0.4375 | 0.2188 | -0.2188 |
| 9 | 0 | Quasi both | 0.9375 | 0.8125 | 0.1250 | 0.0625 | 0.0000 | -0.0625 |
| 10 | 1 | Quasi both | 0.9688 | 0.7188 | 0.2500 | 0.0313 | 0.5313 | 0.5000 |
| 11 | 1 | Quasi both | 0.6875 | 0.2188 | 0.4688 | 0.4063 | 0.6875 | 0.2813 |
| 12 | 0 | Global AP | 0.9063 | 0.9688 | -0.0625 | 0.4063 | 0.0313 | -0.3750 |
| 13 | 1 | Quasi both | 0.5313 | 0.2500 | 0.2813 | 0.2500 | 0.4063 | 0.1563 |
| 14 | 1 | Quasi ob | 0.5313 | 0.1875 | 0.3438 | 0.5313 | 1.9063 | 1.3750 |
| 15 | 1 | Quasi both | 0.9063 | 0.6563 | 0.2500 | 0.0625 | 0.0313 | -0.0313 |
| 16 | 0 | Quasi both | 0.2500 | 0.2500 | 0.0000 | 0.4688 | 0.9688 | 0.5000 |
| 17 | 0 | Global AP | 0.9688 | 1.0000 | -0.0313 | 0.0313 | 0.7500 | 0.7188 |
| 18 | 0 | Global AP | 1.0000 | 0.9375 | 0.0625 | 0.0000 | 0.0938 | 0.0938 |
| 19 | 1 | Quasi ob | 0.4688 | 0.0000 | 0.4688 | 0.7813 | 2.0000 | 1.2188 |
| 20 | 1 | Quasi both | 0.6875 | 0.2188 | 0.4688 | 0.0313 | 3.0625 | 3.0313 |
| 21 | 0 | Quasi both | 0.3750 | 0.2813 | 0.0938 | 0.7500 | 0.1875 | -0.5625 |
| 22 | 0 | Quasi both | 0.3125 | 0.2188 | 0.0938 | 0.2500 | 1.2500 | 1.0000 |
| 23 | 0 | Quasi ob | 0.2188 | 0.1250 | 0.0938 | 0.4063 | 0.5313 | 0.1250 |
| 24 | 1 | Quasi both | 0.7813 | 0.4375 | 0.3438 | 0.1875 | 0.3750 | 0.1875 |
| 25 | 0 | Quasi pno | 0.1875 | 0.2188 | -0.0313 | 1.6875 | 4.0313 | 2.3438 |
| 26 | 1 | Quasi ob | 0.4063 | 0.0938 | 0.3125 | 0.6875 | 0.9688 | 0.2813 |
| 27 | 0 | No quasi | 0.1875 | 0.0625 | 0.1250 | 0.7500 | 1.2813 | 0.5313 |
| 28 | 0 | Quasi both | 0.7188 | 0.8438 | -0.1250 | 0.6875 | 0.7500 | 0.0625 |
| 29 | 0 | No quasi | 0.1250 | 0.0625 | 0.0625 | 1.5313 | 1.1563 | -0.3750 |
| 30 | 0 | No quasi | 0.1875 | 0.1250 | 0.0625 | 1.7188 | 2.1250 | 0.4063 |
| 31 | 0 | Global AP | 1.0000 | 1.0000 | 0.0000 | 0.0000 | 0.0000 | 0.0000 |
| 32 | 0 | No quasi | 0.1875 | 0.0938 | 0.0938 | 1.6875 | 0.4375 | -1.2500 |
| 33 | 0 | Quasi both | 0.5313 | 0.4063 | 0.1250 | 0.5313 | 1.7500 | 1.2188 |
| 34 | 1 | Quasi ob | 0.3125 | 0.0313 | 0.2813 | 0.1250 | 2.3438 | 2.2188 |
| 35 | 0 | Quasi both | 0.7500 | 0.7500 | 0.0000 | 0.1563 | 1.0938 | 0.9375 |
| 36 | 0 | Quasi both | 0.6875 | 0.4375 | 0.2500 | 0.2500 | 0.3125 | 0.0625 |
| 37 | 1 | Quasi ob | 0.6563 | 0.1875 | 0.4688 | 0.1875 | 0.7500 | 0.5625 |
| 38 | 0 | Quasi ob | 0.2188 | 0.1563 | 0.0625 | 0.0313 | 1.5938 | 1.5625 |
| 39 | 0 | Quasi ob | 0.3125 | 0.1250 | 0.1875 | 0.5938 | 1.9688 | 1.3750 |
| 40 | 1 | Quasi both | 0.5313 | 0.2500 | 0.2813 | 1.1875 | 2.7188 | 1.5313 |
|  |  | **M** | **58.594** | **40.703** | **17.891** | **0.4883** | **1.0305** | **0.5422** |
|  |  | ***SD*** | ***28.822*** | ***32.580*** | ***16.338*** | ***0.4791*** | ***0.9356*** | ***0.8462*** |
| ***Notes.*** *In order to preserve participant anonymity, age and gender were not included in this table.* | | | | | | | | |
