# Supplementary material for: Timbral cues underlie instrument-specific absolute pitch in expert oboists
Source: PLoS One. 2024 Oct 3;19(10):e0306974. doi: 10.1371/journal.pone.0306974 (PMC11449301; doi:10.1371/journal.pone.0306974)
Supplement: Attachment — Submitted filename: Response_to_Reviewers.docx [file pone.0306974.s002.docx]

**Response to reviewers**

Please note that in addition to the changes listed below, we noticed that the sub-section titled “Accuracy by pitch” should be placed under “Undeclared Exploratory Tests” rather than under “Declared Exploratory Tests” and updated the manuscript accordingly.

**Reviewer #1:**

I’ll divide my comments into items that I feel need to be fixed before publication to ensure methodological rigor, and those that are just recommendations which I feel could make the paper stronger but aren’t strictly necessary. Since this is a Registered Report (kudos to the authors for using this format), those comments in the former category mainly pertain to the exploratory analyses and discussion. As for those comments in the latter category, I recommend the editor leave it up to the authors’ discretion whether recommendations be implemented.

**Main criticisms:**

------------------------

In Experiment 1, the difference between accuracy on oboe and piano trials is compared for each participant at the nominal significance level of 0.05. One might be concerned about a multiple comparisons problem here, since the probability of finding at least one false positive ISAP subject is greater than 0.721. A quick back-of-the-napkin calculation, assuming that the number of false positives among the a priori maximum n = 50 subjects is drawn from the distribution k ~ Binomial(50, 0.05), indicates that P(k >= 4) = 0.104 but P(k >= 5) = 0.038, so the present results (i.e. that k >= 10) do, in fact, allow you to reject the null hypothesis that there are no ISAP subjects in the population. This is probably worth mentioning in the manuscript, since the statistical inference the authors want to make does technically require such a correction.

We appreciate the reviewer’s attention to this issue and have now added the following text to the Results section of Experiment 1:

“Further analysis addressed concerns for multiple comparisons and for bias in the estimate of ISAP prevalence in the population of oboists in this experiment. We first considered the probability of none of the participants actually having ISAP, given the observations made in the experiment. Assuming the number of false positives is drawn from a binomial distribution, the probability of no oboists actually having ISAP, given that we observed 15 participants with ISAP out of a sample of 40 oboists, is *P*(k >= 15) = .0008. This result supports rejection of the null hypothesis that there were no participants with true ISAP in the tested population.”

The details of the exploratory analysis described under “Modeling Transposition as a

Continuous Variable” are not totally clear in the manuscript. Looking at the R code, however, it seems that while a random intercept for each participant was included in the preregistered analysis (as it should be), it was dropped from this exploratory analysis. Please add the random intercept, or regression parameter estimates may be biased. (This should just be a one-line change to your code, and who knows, you might find something interesting!)

Thanks for noticing this. We have now updated this analysis (in the R code as well as the reporting of it in the manuscript). While the regression parameter estimates do indeed vary slightly, the overall conclusions remain the same as previously reported.

I’m not sure Figure 4 provides strong evidence for a continuum in ISAP (as opposed to just a continuum in AP ability) as the authors suggest in Discussion. While pitch-labeling accuracies for oboe notes do show quite a range in the present sample, they’re also very well correlated with accuracy for piano notes, which presumably reflect gradations of global AP ability. Consequently, much of the variation in ISAP ability might be explained by known variation in AP ability to which performance gains due to instrument-specific timbre cues are “added on.” It’s hard to say just by looking at it, but the drop in performance when timbre cues are disrupted in Experiment 2 seems to actually have very little variance across subjects (i.e. the lines denoting paired differences in Figure 7 mostly have similar slopes). I think that whether the (unique) contribution of instrument-specific cues to pitch-labeling ability is a stable individual difference makes an interesting scientific question for future research, but the present data don’t seem to indicate whether or not this is the case.

We agree that the question of to what extent there is variance in ISAP ability is an intriguing one for future research. We understand Figure 4 as demonstrating potential variance along a continuum, as different participants with ISAP demonstrate a greater or lesser distance from the diagonal, where the diagonal here represents equal performance on piano and oboe. Differences in global AP ability cannot explain this variance in ISAP ability. Because we have not quantified this variance in the paper, and we recognize that interpretations of variance based on visuals (Figs. 4 and 7) are subjective, we have softened our language on this topic and added text suggesting that this question should be investigated in future work.

**Other recommendations:**

-------------------------------------

In the same vein as my comment re: multiple comparisons in Experiment 1 above, ignoring the possibility of Type I & II errors at the subject level will result in a biased estimate of population prevalence. Fortunately, this is relatively simple to remedy if you’d like. If you either establish some minimum effect size of interest for the subject-level chisquared test (i.e. for oboe vs. piano accuracy) and then compute power for that effect size or just assume a power of 1 for a conservative lower bound, then population prevalence can be estimated directly from assumed power and the subject-level p-values using a simple Bayesian calculation (see Ince et al., https://doi.org/10.7554/eLife.62461). R functions for computing these population prevalence estimates, as well as differences in prevalence between conditions (e.g. in AP ability between piano and oboe conditions), with helpful example scripts, can be found here (<https://github.com/robince/bayesianprevalence>). This is not a necessary addition, but could strengthen the paper; just keep in mind that “population prevalence” refers to the population sampled, rather than the general population.

Thank you for bringing our attention to this relevant work. We have used the R package and report the results in the Results section, text copied below:

“Second, to address bias in the observed population prevalence (0.375), we used a Bayesian method [40] to estimate population prevalence from the results of our within-participant tests (*bayesian-prevalence* package in R). Given the observations in Experiment 1, the maximum a posteriori (MAP) estimate—the proportion of the population we would expect to be a true positive for ISAP—is 0.34, (95% HPDI: [0.20 0.50]). This is close to our observed prevalence of 0.375. Here, the highest posterior density interval (HPDI) is a measure of uncertainty, akin to a confidence interval; that is, the probability that the population prevalence is higher than 0.20 is greater than 95%.”

As noted above, I do not think the current data provide specific evidence for a continuously grated distribution of subject-level effect sizes for instrument-specific timbre cue contributions to pitch-labeling ability as the authors suggest. It is worth noting, however, that previous research arguing that AP ability is continuously distributed (e.g. https://doi.org/10.1371/journal.pone.0244308) has been criticized for using complex rather than sine tones, precisely because of the concern that the use of timbre cues could confound “true” – or in the terminology of the present authors, “global” – pitch-labeling ability. (Full disclosure: I am an author on the linked paper, though it was already cited by the present authors.) The results of Experiment 2 seem to indicate that removing/disrupting timbre-cues reduces but does not destroy pitch-labeling accuracy, leaving substantial between-subject variance remaining. This study is a nice contribution to the literature in that regard, as it seems to falsify the above criticism.

We appreciate the reviewer’s observation and agree about the contribution of the results from Experiment 2.

Please see our response to Point #3 regarding a continuum in ISAP ability.

It would be helpful to visualize the rejection threshold for labeling someone as ISAP (as well as lines for at-chance and for the threshold that defines above-chance performance) in Figure 4, rather than just coloring the points.

We appreciate these suggestions and have updated our Figure 4 and the corresponding figure legend, copied below:

“**Figure 4.** Mean accuracy (proportion correct) for oboe (ob) and piano (pno) tones for each of the 40 participants in Experiment 1 with color indicating ISAP status and symbol shape indicating membership of the subtypes included in Table 2. Grey dots represent all hypothetical values for which oboe performance would qualify as significantly better than piano performance (ISAP; bottom right) or vice versa (top left). The dotted lines show thresholds for above-chance performance for piano (horizontal) and oboe (vertical).”

It is interesting that pitch-labeling accuracy, but not precision (were we to accept the null), is affected by the pitch shift, seeing as Fig. 6 indicates responses are non-random on incorrect trials generally. I’d think that, for example, if you pitch-shifted an A note to become a B with the timbre of an oboe-A, the timbre cues would bias the subject to respond something closer to A (or just “false alarm” respond A more often) than they would otherwise. Does the direction of the (not-absolute) semitone error depend on the direction of the pitch-shift? Or are subjects more likely to respond with the note with the original timbre? I think this would be interesting to add, but of course not necessary.

Thank you. We have conducted further descriptive and inferential statistical analysis to answer the reviewer’s two questions.

With regard to the first question (Does the direction of the semitone error depend on the direction of the pitch shift?), it is indeed the case that if the note was pitch-shifted up, participants tended to underestimate, or guess a lower pitch, whereas if the note was pitch-shifted down, participants tended to overestimate, or guess a higher pitch. That is, the bias was in the direction of the original pitch. A chi-squared test with continuity correction found that these differences were significant (*χ^2^*(1) = 16.456, *p* = .00004979). We have now added text regarding this analysis under the section “Undeclared Exploratory Tests” as well as added text regarding its interpretation as converging evidence in the Discussion section.

With regard to the second question (*Are subjects likely to respond with the original note?*), we compared the number of incorrect pitch-shifted trials in which the participant response matched the original note (45) and the number of incorrect pitch-shifted trials in which the participant response did not match the original note (174), showing that 20.5% of the time, participants guessed the original pitch (i.e., the one prior to pitch-shifting). It is tricky to interpret whether this result confirms the reviewer’s expectation or not: it is above the random chance level of 8.33% (i.e., assuming that all responses are equally likely); however, we suspect the actual likelihood of guessing the original note is higher, considering potential cues of register and the data in Figure 6. Because of the difficulty of choosing a meaningful threshold here for interpretation, we have not reported our response to the reviewer’s second question in the paper.

I would love to see more discussion of how it is mechanistically possible for accuracy to be reduced without a corresponding increase in mean absolute semitone deviation. I'm guessing something interesting may be going on here along the lines of my above comment, but maybe the authors have other ideas.

We agree with the reviewer that this is an interesting question. At the moment, we do not have a convincing theory and would therefore also be very curious to see what future research may reveal about this.

P.S. to the authors: I recommended "major revisions" since some of these changes could be substantive (depending on which suggestions you choose to implement or whether adding a random intercept changes results). However, I mentioned in my private note to the editor that revisions per my comments could end up being pretty minor. In my opinion, this manuscript is near the finish line.

We are thankful for the reviewer’s very constructive comments, and we agree that implementing the majority of their suggestions has significantly improved our manuscript.

**Reviewer #2:**

The study defines “instrument-specific absolute pitch” (ISAP) as a “gain in absolute pitch identification ability for one’s own instrument type,” and the possessors of a “global absolute pitch” (AP) as those who can identify pitches “with high accuracy across a range of timbres.” Bachem’s (1955) seminal work is missing here, since he had already pointed out this phenomenon he named “quasi” AP.

Thanks for the suggestion. We now include a reference to Bachem (1955).

In our understanding, Bachem (1955) defines quasi-AP in the following way: “types of absolute pitch based upon a comparison of tones with a standard, the terms quasi-absolute pitch may be proposed.” Revisiting Bachem’s text, we did not actually find that he was referencing the specific phenomenon that we describe as ISAP at any point. Bachem’s definition of quasi-AP is the use of relative pitch in conjunction with a memorized reference tone. Because that strategy should work equally well with any timbre, it is different from ISAP (as we describe in our Discussion section). Our use of the term “quasi-AP” follows Wilson et al.’s (2009) terminology, which is why we cite this work.

Between the lines 87-91, the group identified as AP possessors are those who performed above 85% in identifying correct pitch stimuli. The references provided have measured the AP construct in different ways (i.e., duration of stimuli, percentage of correct responses, timbres), although two of them share commonalities (i.e., only correct pitches are considered for AP). Later, between lines 113-115, another work (Li [20]) is cited. According to Li’s work, “73% correct without semitone errors and 80% with semitone errors” are considered for AP’s ability. Reference [10] provides AP assignment that includes semitone errors. Therefore, AP phenomenon continue to be arbitrarily defined, and it is necessary to clarify why AP possessors in this work are considered those whose correct responses are above 85%. Why wouldn’t it be arbitrary if there is no consensus in the literature? The issue should be further explored and justified. In addition, if there is no consensus about the underlying factor for AP, it is even harder to affirm that ISAP is an existent cognitive ability, and the fact the participants were not sure about they possess it or not might be due to the lack of clarity of the construct model, even if they affirm that they are aware of its existence.

Note that in defining what we refer to as “global AP”, we used a threshold of 90% accuracy. This criterion was taken from Wilson et al. (2009) who, in turn, seem to have adopted it from Takeuchi and Hulse (1993). It is true that in our Introduction section, we used an arbitrary value of “~85%” to exemplify. As we see that references to two different thresholds can be confusing to the reader, we have now changed the value to 90% in the Introduction as well.

As we understand it, Li et al did not define AP as 73% or 80%. Rather, these were the measured performance levels of those of their participants who had started music training at 9 years of age or younger. The authors seem to accept these levels as AP ability, but thresholds for AP are not explicitly stated.

We agree with the reviewer that definitions of AP are not consistent throughout the literature and that the used cut-offs (e.g., 80%, 85%, or 90%) are indeed somewhat arbitrary. In this context, the best we can do is to choose a common cut-off (i.e., that of Wilson et al., 2009, and Takeuchi & Hulse, 1993) and to declare this decision clearly. To this end, we have now added the following text to our manuscript: “While there is no single, agreed-upon threshold for global AP, we followed [13] in considering semitone errors as incorrect responses and categorizing participants scoring >90% correct as possessors of global AP.”

Finally, we would like to draw attention to the fact that our definition of ISAP does not depend on the definition of global AP – i.e., to qualify for ISAP, participants still need to perform significantly better on their own instrument. Therefore, the lack of a consensus on how to define AP does not have any bearing on whether ISAP is an existent cognitive ability or not.

At lines 214-216, on what grounds are the authors relying to support the claim that the accuracy is above chance level?

Thanks for noticing this missing piece of information. As described in the Registered Reports Protocol, this assessment relied on a statistical procedure previously used by Reymore and Hansen (2020, *Front Psychol*): “Specifically, the first criterion for ISAP, above-chance performance for oboe tones (i.e., accuracy > 8.3%), will be assessed with a one-sample proportions test with continuity correction” (Hansen & Reymore, 2021, *PLOS ONE*).

To clarify this in in the current document, we have now re-introduced text from the “Experimental design & statistical analysis” section of the Registered Reports Protocol into the Registered Reports Research Article: “Following our Registered Reports Protocol [36], these hypotheses were tested using a one-sample proportions tests with continuity correction and Pearson’s chi-squared tests with Yates’ continuity correction.”

At line 221, describe how recruitment was done.

We have added the following text to the paper: “Participants were recruited online through Facebook groups for oboists/double reed players and the International Double Reed Society. Email invitations to participate were also sent individually to professional oboists known to one of the authors, who is an oboist.”

At lines 252 – 256, it seems that the authors are not considering the high level of ear training experience the participants have. In other words, the possibility that they made use of the relative pitch memory is not irrelevant (what was indeed declared at the self-report questionnaire by 28 participants out of 40 [line 440] after the tests). This is especially true considering that the participants could listen to the stimuli as many times as they want to.

We agree with the reviewer that the use of relative pitch ability is an important issue to consider. We discussed this topic at length in the Discussion section of the original manuscript:

“The prominence of relative pitch as a strategy among both ISAP and non-ISAP oboists also merits discussion. Of course, relative pitch cannot be used successfully without the presence of a known reference pitch. As we previously argued [36], it is possible that individuals may have memorized a particular pitch, such as the tuning ‘A4,’ and are able to auralize and use this pitch as a reference pitch (as was also proposed by Bachem in 1955)—however, we note that this strategy should likely work equally well with any timbre. In Experiment 1, the strategic use of such an anchor note was mentioned by seven participants. However, none of those participants exhibited ISAP. Participants who specifically described the use of relative pitch also mentioned other strategies which could plausibly be linked to finding a reference tone, including recognition based on instinct/familiarity or on association with a piece with a known starting pitch. Previously [36], we remarked that timbrally-informed identification of a particular note from the primary instrument may result in ISAP via a mixture of instrument-specific absolute and relative pitch identification strategies. In general, participants described multiple strategies, and relative pitch was never listed as a participant’s only strategy. This suggests that many oboists may use a mix of absolute and relative pitch strategies, where ISAP provides an advantage as a source of reference tones, for example, via timbrally idiosyncratic pitches on the instrument in question.”

Between lines 259-264, reference [12] does not provide a validated tested model for ISAP.

We are not sure what the reviewer is referring to here. Reference [12] is a case study, and this is how we describe it in ll. 259-264 of the original manuscript. We agree with the reviewer that observations made in case studies cannot be generalized, but we also do not believe that we make such a claim.

At Figure 5, the differences between the two groups in performance in both white and black keys are very slight: how is the grouping justified here so as not to appear arbitrary?

We take this comment to mean that the reviewer is not convinced either (1) that an analysis comparing between pitches associated with black and white piano keys is justified as more than an arbitrary division or (2) that an analysis comparing participants with and without ISAP is justified as more than an arbitrary division.

To the former, we would like to point out that the grouping between notes associated with black and white piano keys is ultimately an approximation of rarely (black) vs. frequently (white) occurring notes in Western art music, as we also explain in the manuscript. These probability patterns historically relate to pre-equal-temperament tunings of keyboard instruments. This approximation is commonly used in AP research by others (e.g., Simpson & Huron, 1994; Dohn et al., 2014; Miyazaki, 1989, 1990) and by us (Reymore & Hansen, 2020). In response to the latter concern, ISAP is clearly defined elsewhere in our manuscript, and the division between people with and without this ability is a core premise for our work.

The numerical differences in accuracy – between black and white keys and between participants with and without ISAP – may seem subtle from the graph. Yet, the presence of statistically significant main effects of key color and group in themselves justify that this is worth addressing in an exploratory analysis.

At lines 406-406, pitch confusion is discussed. In this case, it is very possible that relative pitch is being used by participants. This possibility should be considered here.

Regarding the use of relative pitch cues, please see our response to the previous comment by the reviewer addressing this topic. We agree with the reviewer that this topic merits discussion, but we prefer to include this in the Discussion section rather than in this part of the Results section.

Line 486 mentions the Registered Report to refer to the simulations to determine the minimum sample size. I suggest it is briefly provided in this paper.

We appreciate the reviewer’s concern for readability. We have included a phrase to identify where the data for the simulations are coming from in the Participants section of Experiment 1 and Participants and Procedure in Experiment 2. However, the simulations are complex to describe, and the amount of text required to do so in detail would impair the flow and comprehension of the methods section in the current manuscript.

The same happens in lines 441- 443: I suggest that the possible interferences of the strategies referred to in another article are summarized here.

Because we discuss this topic in further detail in the Discussion, we see that it is better to refer to the Discussion section of the current manuscript rather than to the Registered Reports Protocol, and the section now includes a line that reads: “This will be considered in more detail in the Discussion below.”

At the Conclusions, at line 815, I would recommend the use of “The current study suggests that etc.”, instead of “The current study provides the first evidence etc.”

We have modified the text accordingly.

In the same way, at line 664-665, I would recommend: “our data suggest,” instead of “our data confirm.”

We have softened our language by using the phrase “our data are consistent with.”

Also, at line 666, the reference 47 refers to a study supposedly conducted with non-musicians according to the text, but only musicians were tested there.

Thanks for noticing this mistake. We now refer to these references together as evidence for a continuum of pitch-identification ability.

The findings and the discussions about pitch shifting described at p. 14–15 are quite interesting, and it offers important clues to potential strategies used by instrumentalists in pitch perception, but maybe not particularly to AP.

At the final references, n. 42 and 43 should be merged.

Thanks for noticing this. It has now been corrected.
